# postQTL: a QTL mapping R workflow to improve the accuracy of true positive loci identification

**DOI:** 10.1186/s13104-022-06017-z

**Published:** 2022-05-04

**Authors:** Prashant Bhandari, Tong Geon Lee

**Affiliations:** 1 Horticultural Sciences Department, University of Florida, Gainesville, FL 32611 USA; 2 Gulf Coast Research and Education Center, University of Florida, Wimauma, FL 33598 USA; 3 Plant Breeders Working Group, University of Florida, Gainesville, FL 32611 USA; 4 Plant Molecular and Cellular Biology Graduate Program, University of Florida, Gainesville, FL 32611 USA

**Keywords:** QTL, Mapping, Model search, Regularization, R workflow

## Abstract

**Objective:**

The determination of the location of quantitative trait loci (QTL) (i.e., QTL mapping) is essential for identifying new genes. Various statistical methods are being incorporated into different QTL mapping functions. However, statistical errors and limitations may often occur in a QTL mapping, implying the risk of false positive errors and/or failing to detect a true positive QTL effect. We simulated the power to detect four simulated QTL in tomato using cim() and stepwiseqtl(), widely adopted QTL mapping functions, and QTL.gCIMapping(), a derivative of the composite interval mapping method. While there is general agreement that those three functions identified simulated QTL, missing or false positive QTL were observed, which were prevalent when more realistic data (such as smaller population size) were provided.

**Results:**

To address this issue, we developed postQTL, a QTL mapping R workflow that incorporates (i) both cim() and stepwiseqtl(), (ii) widely used R packages developed for model selection, and (iii) automation to increase the accuracy, efficiency, and accessibility of QTL mapping. QTL mapping experiments on tomato F_2_ populations in which QTL effects were simulated or calculated showed advantages of postQTL in QTL detection.

**Supplementary Information:**

The online version contains supplementary material available at 10.1186/s13104-022-06017-z.

## Introduction

Quantitative trait loci (QTL) affect the phenotypic variation observed in quantitative characters. Quantitative traits (character) display a continuous distribution of phenotypes, and many agronomically important traits (e.g., yield) belong to quantitative traits. Therefore, QTL mapping, the determination of the location of QTL, is one of the first steps to build a scientific basis for plant genetics and breeding.

Various statistical methods have been used for QTL mapping (e.g., Analysis of variance (ANOVA) [1], interval mapping (IM) [2], composite interval mapping (CIM) [3–6], multiple interval mapping (MIM) [7], Bayesian methods [8], and genetic algorithms [9]). Among these, the CIM method, which uses genetic marker covariates to detect QTL, is widely adopted in plants for QTL models [10]. Several more recent derivates of the original CIM are also available (e.g., inclusive composite interval mapping (ICIM) [11] and genome-wide composite interval mapping (GCIM) [12, 13]).

Broman and Speed [14] stated that the key problem with CIM is the choice of the set of markers to use as regressors, inevitably affecting the power for QTL detection (e.g., increasing the variance of the logarithm of the odds (LOD) score). Likewise, QTL mapping is a model selection problem closely concordant with controlling the inclusion of extraneous loci while identifying as many true QTL as possible [14, 15]. While an exhaustive model search would be the ideal solution to the problem, dramatically increasing computational demands by consolidating different models/large genotypic datasets are less clearly advantageous to non-specialist researchers such as plant breeders. In QTL mapping, forward selection or some version of stepwise selection is commonly used because of its computational efficiency and a close approximation of the best subset selection [14–16]. Recently, an R/qtl function stepwiseqtl() with a forward/backward stepwise search algorithm was implemented in the R/qtl package (note stepwiseqtl() was added to R/qtl version 1.09 and later [15]).

Regularization methods (e.g., minimax concave penalty (MCP) [17], least absolute shrinkage and selection operator (LASSO) [18]), have been used in statistics and machine learning for feature selection (e.g., adopted in genomic prediction [19, 20]). Practical implications of such methods for QTL mapping would help us to select a true QTL by distinguishing a true QTL effect from extraneous loci-driven noise (e.g., regression coefficients for a given marker shrink to near 0 where a model fits).

Given the above information, a comprehensive approach integrating the results from both mapping functions, cim() (note that a version of the CIM method is implemented into the R/qtl) and stepwiseqtl(), and regularization method(s) may be as good as a single function-driven QTL mapping, or an optimal strategy to improve the accuracy of true positive QTL identification. Here, we present postQTL, a QTL mapping R workflow that incorporates (i) both cim() and stepwiseqtl(), (ii) widely used R packages developed for model selection, and (iii) automation to increase the accuracy, efficiency, and accessibility of QTL mapping. postQTL is an R script that executes several R function()s and R packages connected by the R language. This package has been developed to be straightforward for use in a typical R environment on Linux/PC hardware. We focus on the tomato, a model fruit crop with relatively large QTL mapping efforts.

## Main text

### Methods

#### Simulating QTL mapping data

In this study, we used the R environment (version 4.1.1; [21]) with the R/qtl package (version 1.50; [22]) on both a stand-alone Windows operating PC and the UF/Research Computing Linux server, HiPerGator 3.0 [23]. A genetic map created for tomato (*Solanum lycopersicum*) [24] was used to simulate two different sized F_2_ populations using the R/qtl function sim.cross(): F_2_ individuals with 1000 (hereafter referred to as population #1) and 100 (hereafter referred to as population #2) to mimic mapping populations or breeding studies. Four simulated QTL were positioned at 5 cM on each of chromosomes 1, 5, 7, and 12, and simulated to have additive effect sizes of 1.0, 0.5, 0.2, and 0.2, respectively (Additional file 1). The missing data percentage was set at 50% and an error rate of 1e^−4^ was used. Three QTL mapping functions, cim(), QTL.gCIMapping() (version 3.3.1; an R function implemented into the GCIM method [25]), and stepwiseqtl() of the R/qtl package, were used to map simulated QTL in both populations #1 and #2. For cim(), covariates 3 and 11 were chosen. For each mapping function, 100 iterations were performed and the identified QTL were reported.

#### postQTL

**Fig. 1:**
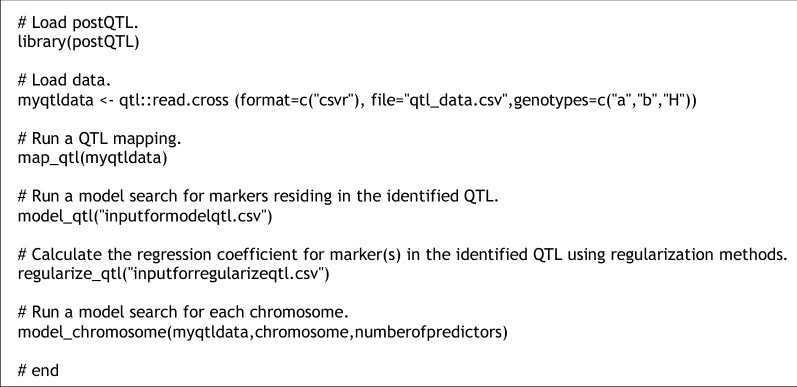
An overview of the postQTL R workflow

The postQTL R workflow developed for this project consists of six steps (Fig. 1; Additional file 2). The first step of the workflow loads the postQTL R script and R packages. Recent versions of commonly used R packages were used for postQTL (the full list of R packages required is available at Additional file 3). The postQTL R script executes four function()s, map_qtl(), model_qtl(), regularize_qtl(), and model_chromosome(). Before executing the script, the user loads an input dataset (a single CSV file that carries the genotypic and phenotypic datasets is available at Additional file 1). After loading, postQTL simultaneously uses cim() with a covariate 11 and stepwiseqtl() to identify QTL (here performed by a function map_qtl()). postQTL performs 10 iterations for cim() and a single run for stepwiseqtl(), and then merges the identified QTL. After identifying QTL, postQTL performs an exhaustive model search for markers residing in the identified QTL using an R function regsubsets() implemented in the R package ‘leaps’ [26] (performed by model_qtl()), providing information about whether or not identified QTL are included in the best model. Such a model search restriction over the identified QTL reduces computational demands. For the QTL identified by stepwiseqtl(), a function find.marker() was used to find the nearest marker to the QTL. For the QTL by cim(), both a marker at the QTL peak position and one of two markers flanking the QTL peak were used. The user determines the best model(s) once a list of models with ranks is created by postQTL [e.g., choose marker(s) with top *Cp* value(s); note that postQTL outputs BIC and adjusted R^2^ using an R package ‘leaps’ as optional additional resources for users). Next, postQTL calculates the regression coefficient (*β*) for the representative marker(s) in the identified QTL using regularization methods LASSO and MCP (performed by regularize_qtl()). LASSO and MCP were implemented using the R packages ‘glmnet’ (version 4.1.3; [27]) and ‘picasso’ (version 1.3.1; [28]), respectively. Finally, postQTL performs an exhaustive model search with a fixed model size (a default value of 3 was set) for each chromosome (e.g., all markers reside on chromosome 1) to identify best predictors (performed by model_chromosome()). Theoretically, a true QTL is more likely to be physically located near or at those predictors. The user can modify arguments listed in postQTL (e.g., chromosome, numberofpredictors) for their QTL mapping project (e.g., a large number of accurate markers vs. a few selected markers). postQTL was tested on a stand-alone Windows operating PC via an RStudio [29] and Linux environment [23] via a terminal emulator.

#### QTL mapping of tomato height

All mapping methods (cim(), QTL.gCIMapping(), and stepwiseqtl()) and postQTL were applied to continuous phenotypic data for the tomato seedling trait. 116 F_2_ plants randomly selected from a segregating progeny in a population, which is known to show at least two QTL (genes) including the tomato *BRACHYTIC* locus [30] for height based on field evaluations, were chosen. The genotypic (F_2_ generation genotyped by the tomato Illumina Infinium array and a molecular marker for the tomato *BRACHYTIC* locus; Additional file 4) and phenotypic (F_2_, F_2:3_, and F_2:4_; Additional file 5) datasets were prepared as described in our previous study [30].

### Results

**Fig. 2:**
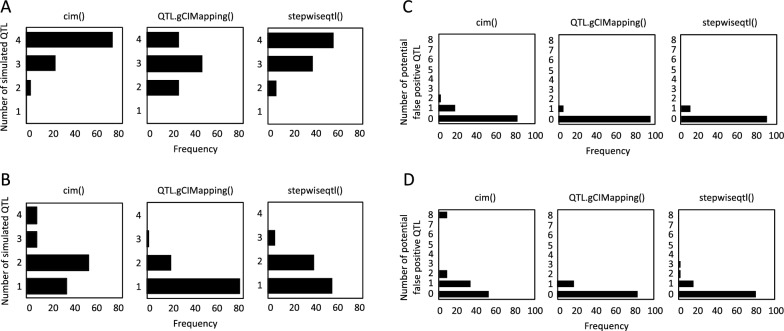
Comparisons of QTL identified by cim(), QTL.gCIMapping(), and stepwiseqtl() for two differently sized populations. Each figure shows the frequency of QTL identified by 100 iterations. **A**, **B** Show simulated QTL (a total of four simulated QTL) identified (LOD > 3.0) for populations of 1000 or 100 F_2_ individuals, respectively (e.g., the left panel in A shows all four simulated QTL were identified by cim() in at least 70 iterations, while two out of four simulated QTL were identified in less than 10 iterations). **C**, **D** Show potential false positive QTL identified (LOD > 3.0) for populations of 1000 or 100 F_2_ individuals, respectively

We simulated four QTL with different effect sizes on four different chromosomes. We compared the QTL mapping results among cim(), QTL.gCIMapping(), and stepwiseqtl() on two differentially sized populations. In simulated population #1 (1000 F_2_ individuals), cim() exhibited the highest frequency of the identification of all four simulated QTL followed by stepwiseqtl() (LOD score > 3.0; Fig. 2A). However, in simulated population #2 (100 F_2_ individuals), a lower accuracy of the identification of simulated QTL was observed for all three mapping functions (Fig. 2B). Both QTL.gCIMapping() and stepwiseqtl() missed at least one of simulated QTL, while cim() identified all four simulated QTL in at least 8% of 100 iterations. Expectedly, the overall frequency of identifying potential false positive QTL in population #2 was higher than in population #1 (Fig. 2C and D). Therefore, in the following sections, we demonstrate how postQTL performed a QTL mapping and its post-analysis on population #2.

In simulated population #2, the first function of postQTL, map_qtl(), identified seven QTL on chromosomes 1, 2, 3, 4, 5, 7, and 12 (column named ‘Mapping’ in Table 1).

**Table 1:** Detailed output generated by postQTL for a population of 100 F_2_ individuals (population #2)

Chromosome no	Simulated QTL	postQTL						Note
		Mapping		Model search	Regression coefficients		Marker prediction	
		Identified QTL	Markers		LASSO	MCP		
1	Q1 (1.0)^a^	Q1	SL4.0ch01.2197362		− 0.12	0.00		
			SL4.0ch01.2487844		0.34	0.00		
			SL4.0ch01.2691495	SL4.0ch01.2691495	0.81	1.01	SL4.0ch01.2691495	
							SL4.0ch01.40326692	
2	None	Q2	SL4.0ch02.429499		− 0.10	− 0.10		False positive
			SL4.0ch02.13770617		0.14	0		
			SL4.0ch02.14095766	SL4.0ch02.14095766	0.03	0.20		
							SL4.0ch02.8249371	
							SL4.0ch02.8542361	
3	None	Q3	SL4.0ch03.3639459	none	0.00	0.00		False positive
							SL4.0ch03.11275781	
							SL4.0ch03.14433259	
4	None	Q4	SL4.0ch04.18671677	SL4.0ch04.18671677	− 0.37	0.00		False positive
			SL4.0ch04.17073658		0.68	0.34		
							SL4.0ch04.41538629	
							SL4.0ch04.43011116	
5	Q5 (0.5)	Q5	SL4.0ch05.796224	SL4.0ch05.796224	0.16	0.20		
			SL4.0ch05.2349396	SL4.0ch05.2349396	− 4.00	− 0.42		
							SL4.0ch05.3014277	
							SL4.0ch05.4441924	
6	None	None	None	n/a			SL4.0ch06.39779625	
							SL4.0ch06.40466450	
7	Q7 (0.2)	Q7	SL4.0ch07.2934431	SL4.0ch07.2934431	− 0.20	− 0.20		
			SL4.0ch07.1720405		− 0.10	0.00		
							SL4.0ch07.4104019	
							SL4.0ch07.7244193	
8	None	None	None	n/a			SL4.0ch08.15907452	
							SL4.0ch08.16388051	
9	None	None	None	n/a			SL4.0ch09.10567972	
							SL4.0ch09.12706265	
10	None	None	None	n/a			SL4.0ch10.22453627	
							SL4.0ch10.53911516	
11	None	None	None	n/a			SL4.0ch11.36161726	
							SL4.0ch11.45414687	
12	Q12 (0.2)	Q12	SL4.0ch12.7161	SL4.0ch12.7161	0.37	0.33	SL4.0ch12.7161	
			SL4.0ch12.3165252	SL4.0ch12.3165252	− 0.29	− 0.28		
							SL4.0ch12.49390591	

n/a: Not available

^a^Genetic effect

Subsequently, postQTL provided a comprehensive approach integrating the results from an exhaustive model search for identified QTL and regression coefficients, which could provide a reasonable guide for QTL selection. In terms of exhaustive model search, postQTL identified markers located in six identified QTL, except for Q3 (column named ‘Model search’ in Table 1). All four simulated QTL (Q1, Q5, Q7, and Q12) were captured by the top models. In the estimated regression coefficient test, relatively high deviations from the *β* value 0 (i.e., not shrink to near 0) for both LASSO and MCP were observed from at least one of the markers representing the simulated QTL (columns named ‘Regression coefficients’ in Table 1). For Q2 and Q4, either an exhaustive model search failed to produce identical markers to representative marker(s) listed in the QTL, or one of two regression coefficient tests for the representative marker(s) shrunk to near 0. In the case of Q3, the exhaustive model search failed to produce any marker(s) on that chromosome. Additionally, the estimated regression coefficient for the marker SL4.0ch03.3639459 in Q3 was close to 0.00, which gives further information to exclude Q3 from a list of potential true positive QTL.

Finally, postQTL identified the best prediction markers per chromosome by running a model search with a fixed model size of 3 (column named ‘Marker prediction’ in Table 1). Two markers, SL4.0ch01.2691495 and SL4.0ch12.7161, were located in the simulated QTL, Q1 and Q12, respectively. Notably, detection of the marker SL4.0ch12.7161, located in one of the small effect QTL, provided further information to control the inclusion rate of small effect QTL.

To validate that postQTL can be useful to identify potential QTL associated with the plant height trait, we performed genetic mapping analyses using postQTL and previously developed mapping functions, cim(), QTL.gCIMapping(), and stepwiseqtl(). Three QTL signals with significant effects (LOD > 4) were detected from at least two filial generations by at least two analyses: a position near the *BRACHYTIC* fine-mapped on chromosome 1 [30], another position (close to 5.0 Mbp) on chromosome 1 and an approximate 60.0 Mbp on chromosome 7 (Additional file 6). Importantly, postQTL exhibited the QTL signal on chromosome 7 from all three filial generations (F_2_, F_2:3_, and F_2:4_), which indicates this location can be used to investigate desirable alleles that might explain the phenotypic segregation in the tomato population used in this study.

### Discussion

Despite the availability of multiple methods for QTL mapping, a need exists for a comprehensive approach integrating the results from multiple QTL mapping methods, which may be the optimal strategy to most accurately identify QTL. We developed postQTL, an R workflow that implements two widely adopted QTL mapping functions. We used postQTL primarily in a tomato community, as tested on F_2_ and other advanced populations. However, postQTL should apply to any (plant) species as long as the QTL mapping functions, cim() and stepwiseqtl(), fit the species of user interest. In the mapping of low genetic effect QTL, missing such QTL is likely to be observed when researchers repeat the mapping analysis with independent imputations. To address this, postQTL has the default number of iterations for cim() as 10.

A critical element of any QTL mapping workflow is its ease of use. postQTL is suitable for both R environment novices and experienced R users. postQTL automates the entire QTL mapping process by requiring only one R workflow. Further, postQTL only requires commonly used R packages in the R program, not requiring additional processing steps outside of the workflow.

Lastly, postQTL includes regularization methods which could be useful supplements to the researcher when the conclusions on QTL determination can be subject to considerable uncertainty.

## Limitations

postQTL limits an exhaustive model search for markers residing in the identified QTL and for markers for each chromosome. Clearly, an exhaustive model search for all genetic markers incorporated into QTL mapping data should be optimized to maximize computational efficiency.

## Supplementary Information

**Additional file 1. **Input dataset.

**Additional file 2. **PostQTL R script.

**Additional file 3. **List of R packages required.

**Additional file 4. **Genotypic data of 116 tomato individuals.

**Additional file 5. **Phenotypic data of 116 tomato individuals.

**Additional file 6. **QTL mapping of tomato height.

## Data Availability

All data generated or analyzed during this study are included in this published article and its supplementary information files.

## Abbreviations

ANOVA: Analysis of variance
BIC: Bayesian information criterion
CIM: Composite interval mapping
GCIM: Genome-wide composite interval mapping
ICIM: Inclusive composite interval mapping
IM: Interval mapping
LASSO: Least absolute shrinkage and selection operator
LOD: Logarithm of odds
MCP: Minimax concave penalty
MIM: Multiple interval mapping
QTL: Quantitative trait loci

## References

[1] Soller M, Brody T, Genizi A (1976). On the power of experimental designs for the detection of linkage between marker loci and quantitative loci in crosses between inbred lines. Theoret Appl Genet.

[2] Lander ES, Botstein D (1989). Mapping mendelian factors underlying quantitative traits using RFLP linkage maps. Genetics.

[3] Jansen RC (1993). Interval mapping of multiple quantitative trait loci. Genetics.

[4] Jansen RC, Stam P (1994). High resolution of quantitative traits into multiple loci via interval mapping. Genetics.

[5] Zeng Z-B (1993). Theoretical basis for separation of multiple linked gene effects in mapping quantitative trait loci. Proc Natn Acad Sci USA.

[6] Zeng Z-B, Kao C-H, Basten CJ (1999). Estimating the genetic architecture of quantitative traits. Genet Res.

[7] Kao C-H, Zeng Z-B, Teasdale RD (1999). Multiple interval mapping for quantitative trait loci. Genetics.

[8] Satagopan JM, Yandell BS, Newton MA, Osborn TC (1996). A Bayesian approach to detect quantitative trait loci using Markov chain Monte Carlo. Genetics.

[9] Carlborg O, Andersson L, Kinghorn B (2000). The use of a genetic algorithm for simultaneous mapping of multiple interacting quantitative trait loci. Genetics.

[10] Doerge RW (2002). Mapping and analysis of quantitative trait loci in experimental populations. Nat Rev Genet.

[11] Li H, Ye G, Wang J (2007). A modified algorithm for the improvement of composite interval mapping. Genetics.

[12] Wang SB, Wen YJ, Ren WL, Ni YL, Zhang J, Feng JY, Zhang YM (2016). Mapping small-effect and linked quantitative trait loci for complex traits in backcross or DH populations via a multi-locus GWAS methodology. Sci Rep.

[13] Wen YJ, Zhang YW, Zhang J, Feng JY, Dunwell JM, Zhang YM (2019). An efficient multi-locus mixed model framework for the detection of small and linked QTLs in F2. Brief Bioinform.

[14] Broman KW, Speed TP (2002). A model selection approach for the identification of quantitative trait loci in experimental crosses. J R Stat Soc Ser B Stat Methodol.

[15] Broman KW, Wu H, Sen S, Churchill GA (2003). R/qtl: QTL mapping in experimental crosses. Bioinformatics.

[16] Sohil F, Sohali MU, Shabbir J (2013). An introduction to statistical learning with applications in R: by Gareth James, Daniela Witten, Trevor Hastie, and Robert Tibshirani.

[17] Zhang C-H. Penalized linear unbiased selection. Technical Report 2007. Dept. Statistics, Rutgers Univ.

[18] Tibshirani R (1996). Regression shrinkage and selection via the lasso. J R Statist Soc B.

[19] Hayes BJ, Bowman PJ, Chamberlain AJ, Goddard ME (2009). Invited review: genomic selection in dairy cattle: progress and challenges. J Dairy Sci.

[20] Crossa J, Campos Gde L, Pérez P, Gianola D, Burgueño J (2010). Prediction of genetic values of quantitative traits in plant breeding using pedigree and molecular markers. Genetics.

[21] R Core Team. R: A language and environment for statistical computing. 2020. R Foundation for Statistical Computing, Vienna, Austria

[22] Arends D, Prins P, Jansen RC, Broman KW (2010). R/qtl: high-throughput multiple QTL mapping. Bioinformatics.

[23] University of Florida. 2022. HiPerGator. https://www.rc.ufl.edu/services/hipergator.

[24] Bhandari P, Lee TG (2021). A genetic map and linkage panel for the large-fruited fresh-market tomato. J Am Soc Hortic Sci.

[25] Zhang YW, Wen YJ, Dunwell JM, Zhang YM (2019). QTL.gCIMapping.GUI v2.0: an R software for detecting small-effect and linked QTLs for quantitative traits in bi-parental segregation populations. Comput Struct Biotechnol J.

[26] Lumley T. 2020. Leaps: R package version 3.1.

[27] Friedman J, Hastie T, Tibshirani R (2010). Regularization paths for generalized linear models via coordinate descent. J Stat Softw.

[28] Ge J, Li X, Jiang H, Liu H, Zhang T, Wang M (2018). Picasso: a sparse learning library for high dimensional data analysis in R and Python. J Mach Learn Res.

[29] RStudio Team. RStudio: Integrated Development for R. RStudio, PBC, Boston, MA URL http://www.rstudio.com/. 2020.

[30] Lee TG, Hutton SF, Shekasteband R (2018). Fine mapping of the brachytic locus on the tomato genome. J Amer Soc Hort Sci.

[31] Fernandez-Pozo N, Menda N, Edwards JD, Saha S, Tecle IY, Strickler SR, Bombarely A, Fisher-York T, Pujar A, Foerster H, Yan A, Mueller LA (2015). The Sol Genomics Network (SGN)–from genotype to phenotype to breeding. Nucleic Acids Res.

