# Supplementary material for: postQTL: a QTL mapping R workflow to improve the accuracy of true positive loci identification
Source: BMC Res Notes. 2022 May 4;15:153. doi: 10.1186/s13104-022-06017-z (PMC9066766; doi:10.1186/s13104-022-06017-z)
Supplement: Supplementary file 2 — Additional file 2. PostQTL R script. [file 13104_2022_6017_MOESM2_ESM.pdf]

```
# postQTL.R

#' cim() and stepwiseqtl()
#' @param cross A cross object used in QTL mapping.
#' @keywords QTL mapping, exhaustive model search
#' @export
#' @import qtl
#' @import leaps
#' @import data.table
#' @import janitor
#' @import tidyverse
#' @importFrom grDevices dev.off pdf
#' @importFrom graphics par
#' @importFrom utils read.csv write.csv

map_qtl <- function(cross){
set.seed(1)
options(warn=-1)
dat1 <- fill.geno(cross, method="argmax",error.prob=0.0001, map.function=c("kosambi"), min.prob=0.95)
write.cross(dat1, format="tidy", filestem="tidy")
CIM_dat100ind_50missing<- dat1
CIM_dat100ind_50missing <- calc.genoprob(CIM_dat100ind_50missing, step=2.0,off.end=0.0, error.prob=1.0e-4,
map.function="kosambi", stepwidth="fixed")
CIM_dat100ind_50missing <- sim.geno(CIM_dat100ind_50missing, n.draws=64, step=2, off.end=0.0, error.prob=1.0e-4,
map.function="kosambi", stepwidth="fixed")

print("Running CIM")

scan.cim_test1_CIM_dat100ind_50missing <- cim(CIM_dat100ind_50missing, pheno.col=1, map.function="kosambi", window=10,
method="em", imp.method="argmax", n.marcovar=11)
scan.cim_test2_CIM_dat100ind_50missing <- cim(CIM_dat100ind_50missing, pheno.col=1, map.function="kosambi", window=10,
method="em", imp.method="argmax", n.marcovar=11)
scan.cim_test3_CIM_dat100ind_50missing <- cim(CIM_dat100ind_50missing, pheno.col=1, map.function="kosambi", window=10,
method="em", imp.method="argmax", n.marcovar=11)
scan.cim_test4_CIM_dat100ind_50missing <- cim(CIM_dat100ind_50missing, pheno.col=1, map.function="kosambi",  window=10,
method="em", imp.method="argmax", n.marcovar=11)
scan.cim_test5_CIM_dat100ind_50missing <- cim(CIM_dat100ind_50missing, pheno.col=1, map.function="kosambi", window=10,
method="em", imp.method="argmax", n.marcovar=11)
scan.cim_test6_CIM_dat100ind_50missing <- cim(CIM_dat100ind_50missing, pheno.col=1, map.function="kosambi", window=10,
method="em", imp.method="argmax", n.marcovar=11)
scan.cim_test7_CIM_dat100ind_50missing <- cim(CIM_dat100ind_50missing, pheno.col=1, map.function="kosambi", window=10,
method="em", imp.method="argmax", n.marcovar=11)
scan.cim_test8_CIM_dat100ind_50missing <- cim(CIM_dat100ind_50missing, pheno.col=1, map.function="kosambi", window=10,
method="em", imp.method="argmax", n.marcovar=11)
scan.cim_test9_CIM_dat100ind_50missing <- cim(CIM_dat100ind_50missing, pheno.col=1, map.function="kosambi", window=10,
method="em", imp.method="argmax", n.marcovar=11)
scan.cim_test10_CIM_dat100ind_50missing <- cim(CIM_dat100ind_50missing, pheno.col=1, map.function="kosambi", window=10,
method="em", imp.method="argmax", n.marcovar=11)

print(summary(scan.cim_test1_CIM_dat100ind_50missing, lodcolumn=1, threshold=3))
print(summary(scan.cim_test2_CIM_dat100ind_50missing, lodcolumn=1, threshold=3))
print(summary(scan.cim_test3_CIM_dat100ind_50missing, lodcolumn=1, threshold=3))
print(summary(scan.cim_test4_CIM_dat100ind_50missing, lodcolumn=1, threshold=3))
print(summary(scan.cim_test5_CIM_dat100ind_50missing, lodcolumn=1, threshold=3))
print(summary(scan.cim_test6_CIM_dat100ind_50missing, lodcolumn=1, threshold=3))
print(summary(scan.cim_test7_CIM_dat100ind_50missing, lodcolumn=1, threshold=3))
print(summary(scan.cim_test8_CIM_dat100ind_50missing, lodcolumn=1, threshold=3))
print(summary(scan.cim_test9_CIM_dat100ind_50missing, lodcolumn=1, threshold=3))
print(summary(scan.cim_test10_CIM_dat100ind_50missing, lodcolumn=1, threshold=3))
print("Extracting markers from CIM")

summary_cim1 <- (as.data.frame(unclass(summary(scan.cim_test1_CIM_dat100ind_50missing, lodcolumn=1, threshold=3))))[-3]
summary_cim2 <- (as.data.frame(unclass(summary(scan.cim_test2_CIM_dat100ind_50missing, lodcolumn=1, threshold=3))))[-3]
summary_cim3 <- (as.data.frame(unclass(summary(scan.cim_test3_CIM_dat100ind_50missing, lodcolumn=1, threshold=3))))[-3]
summary_cim4 <- (as.data.frame(unclass(summary(scan.cim_test4_CIM_dat100ind_50missing, lodcolumn=1, threshold=3))))[-3]
summary_cim5 <- (as.data.frame(unclass(summary(scan.cim_test5_CIM_dat100ind_50missing, lodcolumn=1, threshold=3))))[-3]
summary_cim6 <- (as.data.frame(unclass(summary(scan.cim_test6_CIM_dat100ind_50missing, lodcolumn=1, threshold=3))))[-3]
summary_cim7 <- (as.data.frame(unclass(summary(scan.cim_test7_CIM_dat100ind_50missing, lodcolumn=1, threshold=3))))[-3]
summary_cim8 <- (as.data.frame(unclass(summary(scan.cim_test8_CIM_dat100ind_50missing, lodcolumn=1, threshold=3))))[-3]
```

```
summary_cim9 <- (as.data.frame(unclass(summary(scan.cim_test9_CIM_dat100ind_50missing, lodcolumn=1, threshold=3))))[-3]
summary_cim10 <- (as.data.frame(unclass(summary(scan.cim_test10_CIM_dat100ind_50missing, lodcolumn=1, threshold=3))))[-3]
mar1 <- vector()
for (i in 1:as.numeric(dim(summary_cim1)[1])){im <- find.marker(cross, chr=as.numeric(summary_cim1[i,1]),
pos=as.numeric(summary_cim1[i,2]))
mar1[i]<- im
}
for (i in 1:as.numeric(dim(summary_cim2)[1])){im <- find.marker(cross, chr=as.numeric(summary_cim2[i,1]),
pos=as.numeric(summary_cim2[i,2]))
mar1[i]<- im
}
for (i in 1:as.numeric(dim(summary_cim3)[1])){im <- find.marker(cross, chr=as.numeric(summary_cim3[i,1]),
pos=as.numeric(summary_cim3[i,2]))
mar1[i]<- im
}
for (i in 1:as.numeric(dim(summary_cim4)[1])){im <- find.marker(cross, chr=as.numeric(summary_cim4[i,1]),
pos=as.numeric(summary_cim4[i,2]))
mar1[i]<- im
}
for (i in 1:as.numeric(dim(summary_cim5)[1])){im <- find.marker(cross, chr=as.numeric(summary_cim5[i,1]),
pos=as.numeric(summary_cim5[i,2]))
mar1[i]<- im
}
for (i in 1:as.numeric(dim(summary_cim6)[1])){im <- find.marker(cross, chr=as.numeric(summary_cim6[i,1]),
pos=as.numeric(summary_cim6[i,2]))
mar1[i]<- im
}
for (i in 1:as.numeric(dim(summary_cim7)[1])){im <- find.marker(cross, chr=as.numeric(summary_cim7[i,1]),
pos=as.numeric(summary_cim7[i,2]))
mar1[i]<- im
}
for (i in 1:as.numeric(dim(summary_cim8)[1])){im <- find.marker(cross, chr=as.numeric(summary_cim8[i,1]),
pos=as.numeric(summary_cim8[i,2]))
mar1[i]<- im
}
for (i in 1:as.numeric(dim(summary_cim9)[1])){im <- find.marker(cross, chr=as.numeric(summary_cim9[i,1]),
pos=as.numeric(summary_cim9[i,2]))
mar1[i]<- im
}
for (i in 1:as.numeric(dim(summary_cim10)[1])){im <- find.marker(cross, chr=as.numeric(summary_cim10[i,1]),
pos=as.numeric(summary_cim10[i,2]))
mar1[i]<- im
}
mar2 <- vector()
for (i in 1:as.numeric(dim(summary_cim1)[1])){im <- lodint(scan.cim_test1_CIM_dat100ind_50missing,
chr=as.numeric(summary_cim1[i,1]), expandtomarkers = TRUE)
mar2[i] <- attributes(im)$row.names[1]
}
for (i in 1:as.numeric(dim(summary_cim2)[1])){im <- lodint(scan.cim_test2_CIM_dat100ind_50missing,
chr=as.numeric(summary_cim2[i,1]), expandtomarkers = TRUE)
mar2[i] <- attributes(im)$row.names[1]
}
for (i in 1:as.numeric(dim(summary_cim3)[1])){im <- lodint(scan.cim_test3_CIM_dat100ind_50missing,
chr=as.numeric(summary_cim3[i,1]), expandtomarkers = TRUE)
mar2[i] <- attributes(im)$row.names[1]
}
for (i in 1:as.numeric(dim(summary_cim4)[1])){im <- lodint(scan.cim_test4_CIM_dat100ind_50missing,
chr=as.numeric(summary_cim4[i,1]), expandtomarkers = TRUE)
mar2[i] <- attributes(im)$row.names[1]
}
for (i in 1:as.numeric(dim(summary_cim5)[1])){im <- lodint(scan.cim_test5_CIM_dat100ind_50missing,
chr=as.numeric(summary_cim5[i,1]), expandtomarkers = TRUE)
mar2[i] <- attributes(im)$row.names[1]
}
for (i in 1:as.numeric(dim(summary_cim6)[1])){im <- lodint(scan.cim_test6_CIM_dat100ind_50missing,
chr=as.numeric(summary_cim6[i,1]), expandtomarkers = TRUE)
mar2[i] <- attributes(im)$row.names[1]
}
```

```
for (i in 1:as.numeric(dim(summary_cim7)[1])){im <- lodint(scan.cim_test7_CIM_dat100ind_50missing,
chr=as.numeric(summary_cim7[i,1]), expandtomarkers = TRUE)
mar2[i] <- attributes(im)$row.names[1]
}
for (i in 1:as.numeric(dim(summary_cim8)[1])){im <- lodint(scan.cim_test8_CIM_dat100ind_50missing,
chr=as.numeric(summary_cim8[i,1]), expandtomarkers = TRUE)
mar2[i] <- attributes(im)$row.names[1]
}
for (i in 1:as.numeric(dim(summary_cim9)[1])){im <- lodint(scan.cim_test9_CIM_dat100ind_50missing,
chr=as.numeric(summary_cim9[i,1]), expandtomarkers = TRUE)
mar2[i] <- attributes(im)$row.names[1]
}
for (i in 1:as.numeric(dim(summary_cim10)[1])){im <- lodint(scan.cim_test10_CIM_dat100ind_50missing,
chr=as.numeric(summary_cim10[i,1]), expandtomarkers = TRUE)
mar2[i] <- attributes(im)$row.names[1]
}

print("Running stepwiseqtl")

hcsnpssr<- calc.genoprob(cross, step=2.0,off.end=0.0, error.prob=1.0e-4, map.function="kosambi", stepwidth="fixed")
step_wise <- stepwiseqtl(hcsnpssr, pheno.col=1, max.qtl=10, covar=NULL,scan.pairs=TRUE,additive.only=TRUE, method=c("hk"),
model=c("normal")
)

print(summary(step_wise))

summary_stepwise <- (as.data.frame(unclass(summary(step_wise,lodcolumn=1, threshold=3))))[-4]
mar3 <- vector()
for (i in 1:as.numeric(dim(summary_stepwise)[1])){im <- find.marker(cross, chr=(summary_stepwise[i,2]),
pos=(summary_stepwise[i,3]))
mar3[i]<- im
}
mar <- unique(c(mar1,mar2, mar3))

print(" Markers for putative QTL:"); print(mar)

pheno <- read.csv("tidy_phe.csv", header=TRUE)
pheno_t <- as.data.frame(t(pheno))
pheno_t <- row_to_names(pheno_t,1)
geno <- read.csv("tidy_gen.csv",  header=TRUE)
geno_t <- as.data.frame(t(geno))
geno_t <- row_to_names(geno_t,1)
names.use <- names(geno_t)[(names(geno_t) %in% mar)]
names.use
geno_subset <- geno_t[, names.use]
int1 <- data.frame(lapply(geno_subset, function(x) {gsub("aa", 0, x)
}))
int2 <- data.frame(lapply(int1, function(x) {gsub("bb", 2, x)
}))
int3 <- data.frame(lapply(int2, function(x) {gsub("ab", 1, x)
}))
int4 <- data.frame(lapply(int3, function(x) {gsub("-", 0, x)
}))
geno_subset_recode <- as.data.frame(int4)
geno_subset_recode_numeric <- cbind(pheno_t,geno_subset_recode)
for (i in 1:dim(pheno_t)[2]){colnames(geno_subset_recode_numeric)[i] <- i
}
write.csv(geno_subset_recode_numeric, file="inputforregularizeqtl.csv")
write.csv(geno_subset_recode_numeric, file="inputformodelqtl.csv")

print("Input files for regularize_qtl() & model_qtl() created. Completed Successfully.")
}

#' An exhaustive model search for markers residing in the identified QTL.
#' @param inputformodelqtl A csv file that was generated as an intermediate output from map_qtl.
#' @keywords QTL mapping, exhaustive model search
#' @export
#' @import qtl
#' @import leaps
```

```
#' @import data.table
#' @import janitor
#' @import tidyverse
#' @importFrom grDevices dev.off pdf
#' @importFrom graphics par
#' @importFrom utils read.csv write.csv

model_qtl <- function(inputformodelqtl) {
dataforleaps <- read.csv(inputformodelqtl, header=TRUE)
dataforleaps2 <- dataforleaps[-1]
p <- dim(dataforleaps2)[2] -1
regfit.full <- regsubsets(as.numeric(X1)~. , data=dataforleaps2, nvmax=p, really.big = TRUE, method=c("exhaustive"))

print(regfit.full)

pdf("cp_bic_adjR2.pdf",18,9)
par(mfrow=c(1,2))
par(oma=c(3,3,3,3))
plot(regfit.full,scale = "bic")
plot(regfit.full,scale = "Cp")
plot(regfit.full,scale = "adjr2")
dev.off()
reg.summary<-summary(regfit.full)
post_leaps <- (as.data.frame(reg.summary[1]))
for (i in 1:as.numeric(dim(post_leaps)[1])){im <- colSums(post_leaps)[i]
  post_leaps$colscore[i] <- im
}
post_leaps2 <- (as.data.frame(reg.summary[1]))
for (i in 1:as.numeric(dim(post_leaps2)[1])){im <- rowSums(post_leaps2)[i]
  post_leaps2$rowscore[i] <- im
}
post_leap_final <- cbind(post_leaps, post_leaps2$rowscore)
lowest <- which.min(reg.summary$cp)
lowest <- which.min(reg.summary$cp)
post_leaps <- (as.data.frame(reg.summary[1]))
draft_result <- as.data.frame(post_leaps[lowest+1,])
draft_result <-as.data.frame(draft_result[colSums(draft_result)==1])

print("Selecting the best combination of markers at QTL:")

draft_result
}

#' Regression coefficients.
#' @param input An intermediate csv output file.
#' @keywords  Regularization
#' @export
#' @import  picasso glmnet boot stats tidyverse data.table
#' @import leaps
#' @import picasso
#' @import glmnet
#' @import data.table
#' @import janitor
#' @import boot
#' @import stats
#' @importFrom grDevices dev.off pdf
#' @importFrom graphics par
#' @importFrom utils read.csv write.csv

regularize_qtl <- function(input){
set.seed (1)
options(warn=-1)
dataforleaps <- read.csv(input, header=TRUE)
dataforleaps[dataforleaps$X == "-"] <- NA
dataforleaps <- na.omit(dataforleaps)
dataforleaps2 <- dataforleaps[-1]
colnames(dataforleaps2)[1] <-"X1"
y <-data.matrix(dataforleaps2$X1)
y <- as.numeric(y)
```

```
x <- data.matrix(dataforleaps2[-1])
out2 = picasso(x,y,method="mcp", gamma = 1.25, prec=1e-4)
(out2$beta[,as.numeric(dim(out2$beta)[2])])
train <- sample (1: nrow (x), nrow (x) / 2)
test <- (-train)
y.test <- y[test]
cv.out <- cv.glmnet(x[train , ], y[train], alpha = 1)
bestlam <- cv.out$lambda.min
fit.lasso <- glmnet(x,y)
cv.lasso <- cv.glmnet(x,y)
lasso_mcp<- as.data.frame(cbind(fit.lasso$beta[,as.numeric(dim(fit.lasso$beta)[2])],
out2$beta[,as.numeric(dim(out2$beta)[2])]))
colnames(lasso_mcp)<- c("beta (lasso)", "beta (mcp)")
lasso_mcp
}

#' An exhaustive model search per chromosome with a fixed model size.
#' @param cross A cross object used in QTL mapping.
#' @param chromosome Each chromosome separately.
#' @param numberofpredictors a size of the model i.e. the number of predictors to include in the model.
#' @keywords QTL mapping, exhaustive model search
#' @export
#' @import qtl
#' @import leaps
#' @import picasso
#' @import glmnet
#' @import data.table
#' @import janitor
#' @importFrom grDevices dev.off pdf
#' @importFrom graphics par
#' @importFrom utils read.csv write.csv

model_chromosome <- function(cross,chromosome,numberofpredictors){
set.seed(1)
options(warn=-1)
dat1 <- fill.geno(cross, method="argmax", error.prob=0.0001, map.function=c("kosambi"), min.prob=0.95)
write.cross(dat1, format="tidy",filestem="tidy")
pheno <- fread("tidy_phe.csv", sep=",", header=TRUE, data.table = FALSE)
pheno_t <- as.data.frame(t(pheno))
pheno_t <- row_to_names(pheno_t,1)
map <- fread("tidy_map.csv", sep=",", header=TRUE, data.table = FALSE)
map_t <- as.data.frame(t(map[-3]))
map_t <- row_to_names(map_t,1)
geno <- fread("tidy_gen.csv", sep=",", header=TRUE, data.table = FALSE)
dat_chr <- as.data.frame(t(geno))
dat_chr <- row_to_names(dat_chr,1)
main_map_geno <- as.data.frame(t(rbind(map_t,dat_chr)))
write.csv(main_map_geno,"main_map_geno.csv")
main_map_geno <- read.csv("main_map_geno.csv",header=TRUE)
for(i in 1: 12)
dat_chr01 <- main_map_geno[main_map_geno$chr==chromosome,]
dat_chr01 <- dat_chr01[-2]
int1 <- data.frame(lapply(dat_chr01, function(x) {gsub("aa", 0, x)
}))
int2 <- data.frame(lapply(int1, function(x) {gsub("bb", 2, x)
}))
int3 <- data.frame(lapply(int2, function(x) {gsub("ab", 1, x)
}))
dat_chr01_recode <- as.data.frame(t(int3))
dat_chr01_recode<- row_to_names(dat_chr01_recode,1)
dat_chr_recode_numeric <- cbind(pheno_t,dat_chr01_recode)
dat_chr_recode_numeric <- na.omit(dat_chr_recode_numeric)
colnames(dat_chr_recode_numeric)[1] <- "phenotype"
write.csv(dat_chr_recode_numeric,"dat_chr_recode_numeric.csv")
dat_chr_recode_numeric <- read.csv("dat_chr_recode_numeric.csv",header=TRUE)
dat_chr_recode_numeric <- dat_chr_recode_numeric[-1]
colnames(dat_chr_recode_numeric)[1] <- "phenotype"
regfit.full <- regsubsets(as.numeric(phenotype)~. ,data=dat_chr_recode_numeric, nvmax=numberofpredictors, really.big = TRUE,
method=c("exhaustive"))
```

```
reg.summary<-summary(regfit.full)
lowest <- which.min(reg.summary$cp)
post_leaps <- (as.data.frame(reg.summary[1]))
draft_result <- as.data.frame(post_leaps[lowest+1,])
draft_result <-as.data.frame(draft_result[colSums(draft_result)==1])

print("The best prediction markers per chromosome:")

draft_result
}
```

```
# map_qlt.Rd
% Generated by roxygen2: do not edit by hand
% Please edit documentation in R/postQTL.R
\name{map_qtl}
\alias{map_qtl}
\title{cim() and stepwiseqtl()}
\usage{map_qtl(cross)
}
\arguments{
\item{cross}{A cross object used in QTL mapping}
}
\description{A function that runs cim() & stepwiseqtl()}
\keyword{QTL}
\keyword{exhaustive}
\keyword{mapping}
\keyword{model}
\keyword{search}

# model_qtl.Rd
% Generated by roxygen2: do not edit by hand
% Please edit documentation in R/postQTL.R
\name{model_qtl}
\alias{model_qtl}
\title{An exhaustive model search for markers residing in the identified QTL}
\usage{model_qtl(inputformodelqtl)}
\arguments{
\item{inputformodelqtl}{A csv file that was generated as an intermediate output from map_qtl}
}
\description{An exhaustive model search for markers residing in the identified QTL}
\keyword{QTL}
\keyword{exhaustive}
\keyword{mapping}
\keyword{model}
\keyword{search}

# regularize_qtl.Rd
% Generated by roxygen2: do not edit by hand
% Please edit documentation in R/postQTL.R
\name{regularize_qtl}
\alias{regularize_qtl}
\title{Regression coefficients}
\usage{regularize_qtl(input)}
\arguments{
\item{input}{An intermediate csv output file.}
}
\description{To calculates the regression coefficient for representative marker(s) in the identified QTL using regularization
methods LASSO and MCP}
\keyword{Regularization}

# model_chromosome.Rd
% Generated by roxygen2: do not edit by hand
% Please edit documentation in R/postQTL.R
\name{model_chromosome}
\alias{model_chromosome}
\title{An exhaustive model search per chromosome with a fixed model size}
\usage{model_chromosome(cross, chromosome, numberofpredictors)
}
\arguments{
\item{cross}{A cross object used in QTL mapping.}
\item{chromosome}{Each chromosome separately to reduce computational demands.}
\item{numberofpredictors}{a size of the model i.e. the number of predictors to include in the model}
}
\description{This function is intended to provide optional additional resources}
\keyword{QTL}
\keyword{exhaustive}
\keyword{mapping}
\keyword{model}
\keyword{search}
```

DESCRIPTION

```
Package: postQTL
Title: postQTL
Version: 1.0
Authors@R: c(
    person(given="Prashant",
    family="Bhandari",
    email="prashantbhandari@ufl.edu",
    role = c("aut","cre")),
    person(given="Tong Geon",
    family="Lee",
    email="tonggeonlee@ufl.edu",
    role = c("aut")))
Description: A QTL mapping R workflow to improve the accuracy of true positive loci identification
License: GPL (>= 3)
Encoding: UTF-8
Roxygen: list(markdown = TRUE)
RoxygenNote: 7.1.2
Imports: picasso, leaps, qtl, tidyverse, glmnet, boot, data.table, janitor, readr, stats, glmnet, boot
Suggests: rmarkdown, knitr
NeedsCompilation: no
Packaged: 2022-01-19 15:58:36 UTC; prashantbhandari
Author: Prashant Bhandari [aut, cre], Tong Geon Lee [aut]
Maintainer: Prashant Bhandari <prashantbhandari@ufl.edu>
```

LICENSE

YEAR: 2022
COPYRIGHT HOLDER: Prashant Bhandari and Tong Geon Lee

```
# NAMESPACE

# Generated by roxygen2: do not edit by hand

export(map_qtl)
export(model_chromosome)
export(model_qtl)
export(regularize_qtl)
import(boot)
import(data.table)
import(glmnet)
import(janitor)
import(leaps)
import(picasso)
import(qtl)
import(stats)
import(tidyverse)
importFrom(grDevices,dev.off)
importFrom(grDevices,pdf)
importFrom(graphics,par)
importFrom(utils,read.csv)
importFrom(utils,write.csv)
```
