# Supplementary material for: postQTL: a QTL mapping R workflow to improve the accuracy of true positive loci identification
Source: BMC Res Notes. 2022 May 4;15:153. doi: 10.1186/s13104-022-06017-z (PMC9066766; doi:10.1186/s13104-022-06017-z)
Supplement: Supplementary file 3 — Additional file 3. List of R packages required. [file 13104_2022_6017_MOESM3_ESM.pdf]

Packages required by postQTL

data.table_1.14.2
glmnet_4.1-3
janitor_2.1.0
leaps_3.1
MASS_7.3-53.1
Matrix_1.3-2
picasso_1.3.1
tidyverse_1.3.1
qtl_1.46-2

List of default R packages

base
datasets
graphics
grDevices
methods
stats
utils
