# Supplementary material for: postQTL: a QTL mapping R workflow to improve the accuracy of true positive loci identification
Source: BMC Res Notes. 2022 May 4;15:153. doi: 10.1186/s13104-022-06017-z (PMC9066766; doi:10.1186/s13104-022-06017-z)
Supplement: Supplementary file 6 — Additional file 6. QTL mapping of tomato height. [file 13104_2022_6017_MOESM6_ESM.pdf]

Additional file 6. QTL mapping of tomato height.

Marker	Chromosome no.	Position[a]	LOD[b]	Filial generation	Mapping method
solcap_snp_sl_15000	1	3.0	7.12, 7.1	F_2	stepwiseqtl(), postQTL
solcap_snp_sl_20426	1	5.3	7.1	F_2	stepwiseqtl()
solcap_snp_sl_20420	1	6.8	6.2	F_2	QTL.gCIMapping()
solcap_snp_sl_18635	1	67.4	7.2	F_2	cim()
solcap_snp_sl_18619	1	68.6	9.4	F_2	postQTL
solcap_snp_sl_14003	3	46.6	5.6	F_2	postQTL
solcap_snp_sl_52568	7	59.6	5.9	F_2	postQTL
solcap_snp_sl_5863	7	59.6	4.1	F_2	cim()
solcap_snp_sl_6292	7	63.1	5.8	F_2	stepwiseqtl()
solcap_snp_sl_21394	8	56.6	3.0	F_2	postQTL
solcap_snp_sl_26417	1	62.3	4.4	$F_{2:3}$	QTL.gCIMapping()
solcap_snp_sl_18635	1	67.4	4.2, 4.5, 114	$F_{2:3}$	cim(), stepwiseqtl(), postQTL
solcap_snp_sl_52568	7	59.6	108.2	$F_{2:3}$	postQTL
solcap_snp_sl_15641	11	0.4	109.0	$F_{2:3}$	postQTL
solcap_snp_sl_20426	1	5.3	7.0	$F_{2:4}$	QTL.gCIMapping()
solcap_snp_sl_20420	1	6.8	6.9	$F_{2:4}$	postQTL
solcap_snp_sl_18634	1	67.7	4.7	$F_{2:4}$	cim()
solcap_snp_sl_18619	1	68.6	4.4, 4.4	$F_{2:4}$	stepwiseqtl(), postQTL
solcap_snp_sl_457	1	71.2	4.7	$F_{2:4}$	QTL.gCIMapping()
solcap_snp_sl_10759	2	50.0	5.5	$F_{2:4}$	postQTL
solcap_snp_sl_36192	2	51.4	2.5	$F_{2:4}$	QTL.gCIMapping()
solcap_snp_sl_20049	2	52.1	3.7	$F_{2:4}$	QTL.gCIMapping()
solcap_snp_sl_14003	3	46.6	3.6	$F_{2:4}$	postQTL
solcap_snp_sl_13958	3	47.0	2.7	$F_{2:4}$	QTL.gCIMapping()
solcap_snp_sl_52568	7	59.6	88.1	$F_{2:4}$	postQTL
solcap_snp_sl_5863	7	59.6	15.4	$F_{2:4}$	QTL.gCIMapping()
solcap_snp_sl_6292	7	63.1	100.0	$F_{2:4}$	cim()
solcap_snp_sl_55514	7	63.5	13.1	$F_{2:4}$	stepwiseqtl()
solcap_snp_sl_7384	8	2.8	3.0	$F_{2:4}$	postQTL

[a]The tomato genome version SL4.0 [31].

[a]Logarithm of the odd.
